# COVID-19 and community healthcare: perspectives from Nairobi’s informal settlements

**DOI:** 10.11604/pamj.supp.2020.35.24532

**Published:** 2020-07-08

**Authors:** Peter Mwangi Kibe, Lyagamula Kisia, Pauline Bakibinga

**Affiliations:** 1 Health and Systems for Health Research Unit, African Population & Health Research Center, Manga Close, Off Kirawa Road, P.O. Box 10787-00100, Nairobi, Kenya

**Keywords:** Coronavirus, urban, slums, Kenya

## Abstract

Urban slums are often characterized by overcrowding, inaccessibility of basic services such as running water and abject poverty. These may affect adherence to COVID-19 containment measures and worsen the effect of the virus on slum residents. We explore the overall practices and impact of the COVID-19 mitigation measures on the lives of Nairobi’s urban poor. This was done through a three-week cycle of telephone interviews with residents, local healthcare providers, religious leaders and key decision makers in two of Nairobi’s slums. As the number of COVID-19 cases increase in Kenya, greater efforts are needed to protect those in environments that make it challenging to implement the containment measures. Collaborative effort is needed to firmly and quickly implement social protections and food security measures, protection against domestic violence, and strengthening response at Level One (community level).

## Commentary

In the past couple of weeks since Kenya confirmed its first COVID-19 positive case, the government has rolled out guidelines and directives to stem the spread of the virus. With the number of cases rising daily, more efforts are needed to protect those who are in environments that make it challenging to implement the containment measures. These include urban slums that are characterized by overcrowding, inaccessibility of basic services such as running water and abject poverty. The government of Kenya, like many other countries, took decisive steps to contain the virus soon after the first positive case was confirmed [1]. Citizens were urged to adhere to social distancing, frequent handwashing, use of hand-sanitizers, and to avoid huge gatherings. These measures were accompanied by a nationwide night time curfew, closure of non-essential services, learning institutions and places of worship, a stricter regulation of the public transport sector and cessation of movement in areas with a high number of positive cases. While these containment measures have been touted as the basis of infection prevention, their implementation, especially in urban slums has been a challenge. This is owing to the deficiencies highlighted above that are the normal characteristic of informal settlements [2]. The stay- at- home directive has made it difficult for many to maintain social distance and the loss of jobs means that incomes for many households have either shrunk considerably or been lost altogether. Moreover, having many people at home for consecutive periods of time puts increased pressure on already-strained sanitation facilities and water supply causing a challenge in frequent handwashing as a preventive measure.

**Approach:** as part of an African Population and Health Research Center (APHRC) project on improving health in slums [3], we conducted a three-week cycle of telephone interviews with residents, local healthcare providers, religious leaders and key decision makers in two of Nairobi´s slums. The main aim of the interviews was to explore the overall practices and impact of the COVID-19 mitigation measures on the lives of Nairobi´s urban poor. The project on improving health in slums study is aimed at developing feasible health delivery models for urban slums and has been conducting stakeholder engagements since 2018. Our engagement targets different stakeholders including key decision makers involved in health service delivery in two slum communities with the aim of triangulating data collected from household surveys, spatial mapping of health facilities and health facility surveys and observations to assess health facility capacity in delivering services to the slum residents. With the onset of the pandemic, a shift in the engagement strategy was required; one that allowed continued engagement but adhered to the government´s directives. Through the weekly phone calls we sought to understand the emerging issues in implementing the COVID-19 measures in the slums by exploring the impact of implementation of COVID-19 measures for the slum residents.

### COVID-19 containment measures practices in the slum communities

***Hand washing:*** residents are trying their best to practice personal protective measures especially handwashing. Local shops and small traders have set up simple handwashing stations next to their businesses for their customers. The local leadership has helped to mobilize partners to set up handwashing stations in busy areas such as local markets and boda boda (motorcycle) terminus. The main challenges with handwashing are s the inconsistent supply of water due to rationing and lack of soap. *“Many people are adhering to the hand washing guidelines. Some hand washing points have been provided by organizations but most people have created their own “Do-it-Yourself” hand washing points at the entry of their homes and at the local shops. The community gets piped water two times a week and tanked water once a week from the city council. On other days people are forced to buy water from vendors.” (Community Worker). “...Some organizations have donated hand washing points and soap within the community. This has made it easier for people to wash their hands regularly but we still need more soap.” (Community Worker)*.

**Use of face masks:** as per public health regulations, residents are wearing masks while in public spaces. However, considering the difficult economic situation, many complain that the cost of masks is too high (Ksh 20 to 50). They are forced to make sacrifices to follow this directive. The biggest concern from a public health perspective is the improper use and disposal of masks. *“Many people have the masks but they use them inappropriately. Some keep them in their pocket while others are hanging them on their necks, others covering the chin while others are holding them in their hands. I think they only have them because it is now illegal not to have masks in public places,”* (Health Representative). *“...people have been forced to buy masks, but many cannot afford them.” (Youth Representative)*.

***Social distancing:*** adhering to social distancing has been a major challenge among the slum communities. This can be attributed to congestion within homes and houses being very close to each other. Slums are also more communal and there is little private space. The situation is worse now because most of the people are no longer going to work and schools are also closed. *“Observation of social distance amongst residents is not easily applicable in the slum especially now that schools have closed and children are not going to school. There is no space for social distance, houses are so close together making it difficult to maintain social distance.” (Religious Leader)*.

**Impact of COVID-19 measures in the community:** the COVID-19 containment measures have led to massive closure of companies and other businesses leading to far reaching effects especially for low income daily wage earners. The loss of income has also affected the small scale traders in the slums due to low purchasing power as a result of the surge in the number of unemployed persons in the slums. Many households which were already poverty stricken have been rendered even more vulnerable and are unable to afford food and to pay rent. *“The number of meals people consume has decreased drastically. Many people are now only having one meal a day. Those who would buy food especially in the evening from the street vendors are no longer able to do so. There is an organization that donated food enough for 100 people but that is obviously not enough with more than 40,000 people in the community.”* (Youth Representative) *“People are getting evicted from their homes every other day. When they leave one place, they look for other housing within the community, but many times since they are still not able to afford the housing they are evicted again,” (Community Worker)*

Social disorder is on the rise as a result of the frustrations from the COVID-19 measures. Cases of risky social behavior in the community such as theft and violence have been reported. This comes with the increased anxiety from the economic shocks of COVID-19 and the uncertainty from the seemingly dark days ahead. The slums social fabric which has for a long time been considered an opportunity for key interventions in slums is at the risk of disintegrating. *“Some youths recently tried stealing food that was meant for the elderly in the community and was being donated by some benefactors in the Indian community” (Religious Leader)*. Increased cases of domestic violence and sexual assault have been reported during this period. This surge could be attributed to women and young girls being trapped in with abusers by stay at home measures. The victims remain in close contact with their abusers and may not get help easily due to the disruption of services. *“Cases of domestic violence have been on the rise. Reports of young girls being sexually abused are becoming common. The perpetrators of these heinous crimes are people they live with either in the same household or in the neighborhood,”* (Health worker).

**Call to Action:** there is a need for positive reinforcement on what they are doing well. However, there are serious glaring concerns that if not addressed early are likely to counter all the efforts and drain the progress in COVID-19 prevention measures. Government and partners should therefore consolidate their efforts fast and firmly to implement the following measures:

*Social protections and food security:* coordinated efforts to ensure that all households within the slums are cushioned against food insecurity and that people are safe from hunger and related harms. Food distribution should target all the slum dwellers and should involve community leaders to allow the community to own the interventions. Stakeholders should further ensure that slum residents are safe from other risks emanating from the measures such as eviction orders and mental frustration leading to social ills. Cash transfers have been recommended as an effective way of protecting communities during COVID-19 [4].

*Protection against domestic violence:* stakeholders need to step up means to keep potential victims safe. Hotline responses and quick action by authorities during the stay home period should be activated to ensure that victims have channels of seeking help.

*Strengthening response at Level One (community level):* increase efforts to support Community Health Volunteers to continue with their routine duties by providing appropriate protective wear. Their link to health facilities is important to make sure that people still receive the care that they need, especially for non-COVID related illnesses. With more support they can also keep sensitizing the community on the importance of adhering to guidelines.
